# Multidisciplinary genomic evaluation reveals adult inborn errors of immunity with rheumatic features

**DOI:** 10.70962/jhi.20250218

**Published:** 2026-08-10

**Authors:** Hiroyuki Baba, Tadashi Hosoya, Taiki Yamaguchi, Takuji Itakura, Hirokazu Sasaki, Natsuka Umezawa, Tetsuya Saito, Naoki Kimura, Ryuji Koike, Masayuki Yoshida, Masaki Shimizu, Hirokazu Kanegane, Shinsuke Yasuda

**Affiliations:** 1 Department of Rheumatology, Graduate School of Medical and Dental Sciences, Institute of Science Tokyo, Tokyo, Japan; 2 Department of Medical Genetics, Institute of Science Tokyo, Tokyo, Japan; 3 Department of Pediatrics, Neonatal and Maternal Medicine, Graduate School of Medical and Dental Sciences, Institute of Science Tokyo, Tokyo, Japan; 4 Department of Child Health and Development, Graduate School of Medical and Dental Sciences, Institute of Science Tokyo, Tokyo, Japan

## Abstract

Multidisciplinary genomic evaluation is increasingly recognized for its diagnostic and therapeutic implications in adults with suspected inborn errors of immunity (IEI) presenting with rheumatic and musculoskeletal disease (RMD) phenotypes. We retrospectively analyzed 50 adults with suspected IEI who underwent genetic testing and were pre-classified into immunodeficiency (ID), autoinflammatory disorders (AID), and non-ID/AID groups. Genetic findings, clinical classification, treatment modifications, and exploratory machine learning analyses were evaluated. A final genetic diagnosis consistent with IEI was identified in 15 patients (30.0%), with the highest yield in the non-ID/AID group (44.4%). Variants were most frequently associated with autoinflammatory diseases (40.0%). Four patients, all initially classified as non-ID/AID, were reclassified based on genetic findings and IUIS classification. Treatment was modified in 12 patients, including eight genetically diagnosed patients and three genotype-driven interventions. Two patients died from disease-related complications. Machine learning analyses provided heterogeneous feature contributions across groups. These findings highlight the utility of multidisciplinary genomic evaluation for refining diagnoses in challenging adult patients.

## Teaser

Baba et al. demonstrate the utility of multidisciplinary genetic evaluation for identifying inborn errors of immunity in adult rheumatology practice. They establish a workflow that improves diagnostic precision and management for patients with atypical rheumatic and musculoskeletal diseases.

## Introduction

Inborn errors of immunity (IEI), formerly termed primary immunodeficiency diseases (PIDs), were initially recognized as disorders predominantly affecting children, characterized by severe and recurrent infections due to underlying genetic defects in immune function (1, 2). However, over the past decade, this concept has evolved substantially (3). IEIs are now recognized as a heterogeneous group of disorders encompassing not only immunodeficiency (ID) but also immune dysregulation syndromes, including autoinflammation, autoimmunity, severe allergy, and susceptibility to malignancy (4, 5). To date, over 500 causative genes have been identified, and novel gene–disease associations continue to be reported annually, underscoring the diagnostic and therapeutic challenges associated with these disorders.

With the expansion of the IEI spectrum, it is now well established that the diagnoses of these disorders are not confined to childhood. Recent epidemiological estimates indicate that over 50% of IEI cases may first present their characteristics after the age of 25, highlighting the clinical importance of adult-onset IEI (6, 7, 8). Moreover, the increasing recognition of late-onset entities—most notably VEXAS (vacuoles, E1 enzyme, X-linked, autoinflammatory, and somatic) syndrome—demonstrated that IEI can emerge de novo even in older individuals, particularly through acquired somatic variants of *UBA1* (9). Compared to pediatric-onset IEI, adult-onset cases often exhibit atypical and gradually accumulating manifestations, along with diverse disease courses, resulting in delayed or missed diagnoses (7, 8, 10, 11).

Importantly, a subset of adult patients with IEI presents primarily with systemic inflammation or autoimmune manifestations—symptoms that mimic rheumatic and musculoskeletal diseases (RMD) (12, 13, 14). Indeed, emerging evidence indicates that these patients initially diagnosed with RMD may carry unrecognized monogenic immune defects (15, 16). This diagnostic ambiguity poses significant clinical risks; for instance, a delayed or incorrect diagnosis of RMD may lead to unwarranted use of immunosuppressive therapies, thereby predisposing patients with unrecognized ID to serious infections or further immune compromise (8, 17, 18, 19, 20, 21, 22, 23, 24, 25). Adding to this complexity, variants in autoinflammatory disease-related genes such as *MEFV* have been reported to modify RMD phenotypes, further underscoring that genetic findings must be interpreted with caution in clinical practice (26, 27, 28, 29). Therefore, physicians, particularly rheumatologists caring for adult individuals, must develop a better understanding of these conditions and collaborate across specialties to integrate genetic findings with clinical phenotypes, enabling timely and accurate diagnoses and management.

Genetic testing now plays a central role in the diagnosis of IEI. Advances in next-generation sequencing enable high-throughput, sensitive analysis of immune-related genes, improving diagnostic yield and facilitating mechanism-based decisions (30, 31). However, the utility of genetic testing in adults with RMD remains poorly characterized. The prevalence of pathogenic IEI-related variants in this population, and their implication for clinical management, have not been systematically investigated. Moreover, no standardized criteria currently exist to guide genetic evaluation in adult rheumatology practice.

To address these clinical questions, we conducted a comprehensive evaluation of the diagnostic yield and clinical impact of multidisciplinary genomic evaluation—including its influence on therapeutic strategy—in adult patients with suspected IEI presenting with RMD. Additionally, we investigated the utility of a structured clinical classification framework and machine learning–based approaches in predicting patients most likely to benefit from genetic evaluation.

## Results

### Patient demographics and group classification

**Figure 1.**
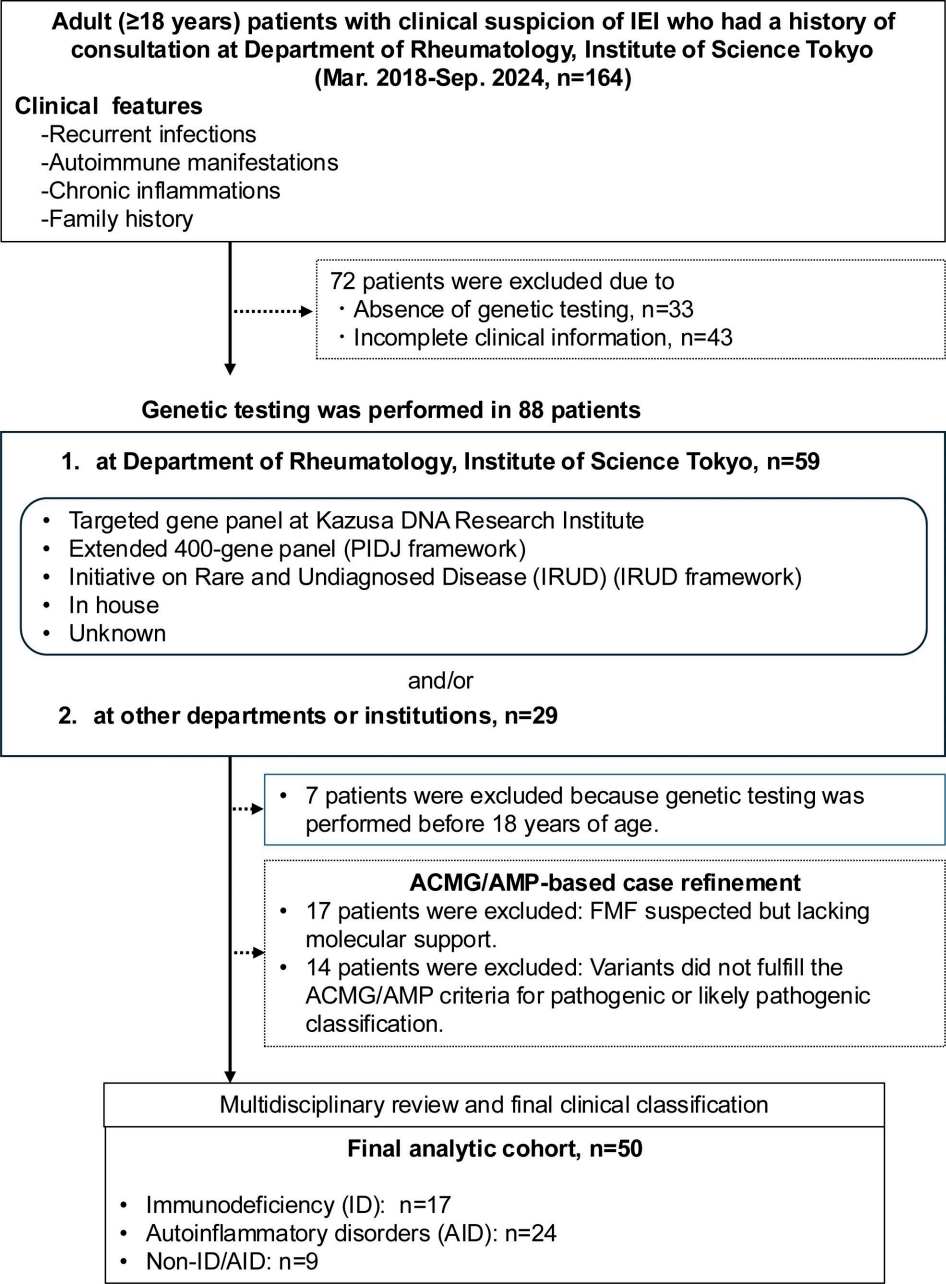
**Study design and clinical classification framework.** Flowchart illustrating the inclusion criteria, classification process, and genetic testing pathways for adult patients with clinical suspicion of IEI. Between March 2018 and September 2024, 164 adult patients presenting with recurrent infections, autoimmune manifestations, chronic inflammation, or suggestive family history were evaluated. After excluding patients without genetic testing (*n* = 33) or with incomplete clinical information (*n* = 43), 88 patients underwent genetic testing. Prior to downstream analyses, 17 patients with clinical familial Mediterranean fever (FMF) lacking molecular support, 14 patients whose identified variants did not fulfill ACMG/AMP criteria for pathogenic or likely pathogenic classification, and 7 patients tested before 18 years of age were excluded. Following result review, the final cohort consisted of 50 adult patients. Patients were classified into three groups—ID (*n* = 17), AID (*n* = 24), and non-ID/AID (*n* = 9)—based on clinical features. Genetic testing was performed using four platforms depending on clinical judgment: (1) targeted panels at KDRI, (2) the expanded 400-gene panel under the PIDJ initiative, (3) the IRUD, and (4) in-house sequencing. A detailed genetic testing workflow and platform-specific information are provided in Fig. S5.

A total of 50 adult patients met the inclusion criteria and were classified into three clinical groups as shown in Fig. 1. The mean age was 36.5 ± 13.8 years, and 68.0% were female (*n* = 34). Based on pretest clinical features, 17 patients were classified as ID, 24 as autoinflammatory disorders (AID), and 9 as non-ID/AID. Patients in the non-ID/AID group were often already diagnosed with RMD such as systemic lupus erythematosus or dermatomyositis (Table S1).

### Baseline characteristics across clinical subgroups

Table 1 summarizes the baseline characteristics across the three groups. The mean age at the time of genetic testing was similar in the ID and AID groups (mean 35.2 ± 8.2 years and 35.0 ± 15.7 years, respectively) but higher in the non-ID/AID group (mean 42.8 ± 16.*5* years). Median disease duration was the longest in the AID group (8.0 years, interquartile range [IQR] 3.0–17.0), followed by non-ID/AID (7.0 years, IQR 1.8–10) and ID (3.0 years, IQR 1.1–23.5). A family history of similar symptoms was most prevalent in the AID group (37.5%), and less common in the non-ID/AID (11.1%) and ID (0%) groups.

**Table 1.** Baseline clinical and demographic characteristics of patients by clinical classification

​	ID (*n* = 17)	AID (*n* = 24)	Non-ID/AID (*n* = 9)
Age at testing, mean (SD)	35.2 (8.2)	35.0 (15.7)	42.8 (16.5)
Age at onset, years (SD)	24.7 (14.7)	23.3 (16.2)	32.6 (21.9)
Disease duration, years, median (IQR)	3.0 (1.1–23.5)	8.0 (3.0–17.0)	7.0 (1.8–10)
Female, *n* (%)	10 (58.8)	19 (79.2)	5 (55.6)
**Family history**	​	​	​
Same disease, *n* (%)	1 (5.9)	3 (12.5)	0
Similar symptoms, *n* (%)	0	9 (37.5)	1 (11.1)
Recurrent infections, *n* (%)	10 (58.8)	0	0
**Purpose of testing**	​	​	​
Differential diagnosis, *n* (%)	15 (88.2)	4 (16.7)	3 (33.3)
Diagnostic clarification, *n* (%)	2 (11.8)	20 (83.3)	6 (66.7)
**Symptoms**	​	​	​
Periodic fever, *n* (%)	0	24 (100)	0
Nonperiodic fever, *n* (%)	4 (23.5)	0	8 (88.9)
Arthralgia/arthritis, *n* (%)	4 (23.5)	15 (62.5)	6 (66.7)
Chest or abdominal pain, *n* (%)	2 (11.8)	11 (45.8)	1 (11.1)
Enterocolitis, *n* (%)	1 (5.9)	8 (33.3)	1 (11.1)
Skin rash, *n* (%)	2 (11.8)	6 (25.0)	4 (44.4)
Headache/meningitis, *n* (%)	0	2 (8.3)	1 (11.1)
Hearing loss, *n* (%)	0	2 (8.3)	0
**Laboratory data**	​	​	​
Lymphocyte count (µl), mean (SD)	1,345 (629)	1,696 (620)	1,004 (476)
Serum IgG levels (mg/dl), median (IQR)	715 (67–914)	1,030 (900–1,261)	1,356 (1,081–1,605)
Autoantibody positivity, *n* (%)	0	2 (8.3)	3 (33.3)
**Pre-genetic treatment**	​	​	​
IVIG, *n* (%)	12 (70.6)	0	0
Colchicine, *n* (%)	0	14 (58.3)	3 (33.3)
Canakinumab, *n* (%)	0	2 (8.3)	0
Glucocorticoids, *n* (%)	3 (17.6)	5 (20.8)	7 (77.8)
Immunosuppressants, *n* (%)	1 (5.9)	6 (25.0)	3 (33.3)

This table summarizes the demographic and clinical features of the 50 adult patients classified into three subgroups based on pre-genetic clinical presentation: ID (*n* = 17), AID (*n* = 24), and non-ID/AID (*n* = 9). Variables include age at genetic testing, sex, disease duration, family history (same disease or similar symptoms), clinical manifestations (e.g., fever pattern, arthralgia, skin rash), laboratory values (serum IgG levels, lymphocyte count, autoantibody positivity), and treatment history prior to genetic diagnosis.

The indication for genetic testing differed by groups: 88.2% of ID cases underwent testing for differential diagnosis, whereas 83.3% of AID patients were tested based on specific clinical suspicion. In contrast, testing in the non-ID/AID group was hypothesis-driven in one-third of patients and exploratory in the remainder.

Clinical manifestations reflected group-specific patterns. Periodic fever was a defining feature of the AID group. Other frequent symptoms included arthralgia (62.5%), chest or abdominal pain (45.8%), and enterocolitis (33.3%). Hearing loss was reported in two patients (8.3%), exclusively in the AID group. The non-ID/AID group exhibited high rates of nonperiodic fever (88.9%) and skin rash (44.4%). Across the 17 patients in the ID group, recurrent infections were observed in 10 patients, among whom upper respiratory tract infections were observed in seven patients (Table S2). Opportunistic and fungal infections occurred only in some patients (*n* = 5 and *n* = 2, respectively). Laboratory findings confirmed absent autoantibodies in ID, low frequency in AID (8.3%), and increased prevalence in non-ID/AID (33.3%), reflecting overlap with autoimmune phenotypes.

Pre-genetic treatment patterns aligned with clinical classification. Immunoglobulin replacement was administered in 70.6% of ID patients, colchicine in 58.3% of AID patients, and systemic glucocorticoids in 77.8% of non-ID/AID patients, indicating an empirical approach to disease management in this diagnostically ambiguous group. In addition, detailed immunological phenotype data were evaluated for patients who received immunoglobulin replacement therapy (Table S3). Serum IgA and IgM levels at the time of genetic testing were frequently low, with IgA below the lower limit of detection in several cases and markedly reduced in more than half of the patients. IgM levels were also decreased in the majority of cases, and a subset of patients exhibited profound hypogammaglobulinemia with both IgA and IgM below detectable limits, consistent with significant impairment of humoral immunity.

### Final diagnoses and categorization based on the genetic findings

A final genetic diagnosis consistent with IEI was identified in 15 of 50 patients (30.0%) based on genetic findings and integrated clinical interpretation (Table 2 and Table S4). The most frequently affected gene was *NLRP3* (*n* = 4), which is associated with cryopyrin-associated periodic syndrome, with all cases confined to the AID group. Other notable findings included haploinsufficiency of *CTLA4* (*n* = 2), *NFKB1* deficiency (*n* = 1), *NFKB2* deficiency (*n* = 1), and *NOD2* (*n* = 1), predominantly in the ID group. Two patients with *UBA1* variants—diagnosed with VEXAS syndrome—were initially classified as non-ID/AID and identified via in-house sequencing. Additional rare variants were found in *PRF1*, *GATA2*, *ALPK1*, and *ICOS*. Based on genetic findings and the International Union of Immunological Societies (IUIS) 2024 phenotypic classification, clinical categories were reclassified in four patients: all were initially classified as non-ID/AID. Following reclassification, patients carrying variants in *UBA1* or *NOD2* were reassigned to the autoinflammatory diseases category, while one patient with *CTLA4* was reclassified to diseases of immune dysregulation. No cases required reclassification from ID-related and autoinflammatory disease categories or vice versa.

**Table 2.** Genetic diagnoses and clinical reclassification before and after genetic testing

Disease	Gene	Total, *n*	Pre-genetic testing classification (*n* = ID, AID, non-ID/AID)	IUIS 2024 group
Cryopyrin-associated periodic syndrome	*NLRP3*	4	0, 4, 0	Autoinflammatory diseases
CTLA4 haploinsufficiency	*CTLA4*	2	1, 0, 1	Diseases of immune dysregulation
NFKB1 deficiency	*NFKB1*	1	1, 0, 0	Predominantly antibody deficiencies
NFKB2 deficiency	*NFKB2*	1	1, 0, 0	Predominantly antibody deficiencies
VEXAS syndrome	*UBA1*	2	0, 0, 2	Somatic-associated autoinflammatory diseases
Blau syndrome	*NOD2*	1	0, 0, 1	Autoinflammatory diseases
Perforin deficiency	*PRF1*	1	1, 0, 0	Diseases of immune dysregulation
GATA2 deficiency	*GATA2*	1	1, 0, 0	Combined immune deficiency
ROSAH syndrome	*ALPK1*	1	0, 1, 0	Autoinflammatory diseases
ICOS deficiency	*ICOS*	1	1, 0, 0	Predominantly antibody deficiencies

This table summarizes the genetic diagnoses identified in the cohort, including associated genes, IUIS disease categories, and corresponding clinical classification before and after genetic testing. The pre-genetic classification was defined prior to genetic testing based on the dominant phenotype: ID, AID, or non-ID/AID. The post-genetic classification reflects the final clinical categorization reassigned after integrating genetic findings and the corresponding IUIS disease group. ROSAH, retinal dystrophy, optic nerve edema, splenomegaly, anhidrosis, headache.

Diagnostic yield was highest in the non-ID/AID group (44.4%). This may reflect the presence of previously unrecognized IEI within this heterogeneous category.

### Therapeutic impact and clinical outcomes

Genetic testing led to therapeutic modifications in 12 of 50 patients (24.0%) overall. Among 15 patients with a final genetic diagnosis, treatment changes were implemented in eight patients (53.3%) following genetic testing. In contrast, 4 of 35 patients without a final genetic diagnosis also underwent treatment modification based on clinical reassessment and phenotype-driven management. Immunoglobulin replacement was newly initiated in five patients—four in the ID group and one in the AID group. Canakinumab, an IL-1β inhibitor, was introduced in two AID cases, and systemic corticosteroids were introduced in one. Immunosuppressive agents were initiated in four patients (AID, *n* = 1; non-ID/AID, *n* = 3), reflecting the heterogeneity of immune dysfunction. Based on integration of clinical context and timing, 3 of the 12 interventions (25.0%) were classified as genotype-driven treatment, primarily corresponding to patients in whom genetic findings directly informed disease-specific therapeutic decisions (Table 3). The remaining nine interventions (75.0%) were considered predominantly phenotype driven, including treatment in patients with common variable ID without an identified monogenic defect, Blau syndrome, or VEXAS syndrome.

**Table 3.** Genotype-driven therapeutic agents administered following genetic testing by clinical subgroup

Added treatment	Final diagnosis	Gene	Pretest classification	Post-test classification
**Immunoglobulin replacemen**t	​	​	​	​
Immunoglobulin	NFKB2 deficiency	*NFKB2*	ID	Predominantly antibody deficiencies
**Autoinflammatory disease–related therapies**	​	​	​	​
Canakinumab	CAPS	*NLRP3*	AID	Autoinflammatory disease
Canakinumab	ROSAH syndrome	*ALPK1*	AID	Autoinflammatory disease

This table summarizes genotype-driven therapeutic interventions newly introduced after genetic testing. Patients are grouped by treatment category, including immunoglobulin replacement and autoinflammatory disease-related therapies. For each case, the final diagnosis, underlying gene, and pre- and posttest clinical classification are presented, illustrating how genetic findings directly informed clinical management.

CAPS, cryopyrin-associated periodic syndrome; ROSAH, retinal dystrophy, optic nerve edema, splenomegaly, anhidrosis, headache.

Two patients died during follow-up due to disease-related complications (Table S5). These included one ID patient with *GATA2* deficiency and one non-ID/AID patient with VEXAS syndrome (*UBA1*). Causes of death were primarily infection-related, despite appropriate prophylactic treatment and extensive antimicrobial treatment. Both patients carried highly pathogenic variants and exhibited complex phenotypes, including pulmonary alveolar proteinosis, myelodysplastic syndrome, and Sweet syndrome, underscoring the clinical severity of adult-onset IEI.

### Comparison between genetically diagnosed and genetically undiagnosed patients

Patients were classified into two groups according to genetic findings: genetically diagnosed, defined as those with pathogenic or likely pathogenic variants consistent with IEI, and genetically undiagnosed, defined as those without such variants. Compared with genetically undiagnosed patients, genetically diagnosed patients more frequently had a family history of the same disease (26.7 vs. 0%, P = 0.005) and less frequently had chest or abdominal pain (0 vs. 40.0%, P = 0.004) (Table 4). Chest or abdominal pain was less frequent (0 vs. 40%, P = 0.004) and lymphocyte counts were lower (mean 1,104/μl vs. 1,566/μl, P = 0.047) in patients with a final genetic diagnosis, while other clinical features were comparable between the groups. Colchicine use was more frequent in patients without a final genetic diagnosis (0 vs. 45.7%, P = 0.002), whereas other treatments did not differ. Moreover, no significant differences were found across ID, AID, and non-ID/AID classification with respect to these clinical variables.

**Table 4.** Comparison of clinical features between patients with and without a final genetic diagnosis

​	With a final genetic diagnosis *n* = 15	Without a final genetic diagnosis *n* = 35	P value
Age at testing, mean (SD)	42.8 (16.6)	33.8 (11.7)	0.057
Age at onset, mean (SD)	26.4 (20.5)	25.0 (15.4)	0.935
Disease duration, years, median (IQR)	9.0 (2.8–35.5)	7.0 (1.5–14.0)	0.210
Female, *n* (%)	8 (53.3)	26 (74.3)	0.191
**Family history**	​	​	​
Same disease, *n* (%)	4 (26.7)	0	0.005
Similar symptoms, *n* (%)	5 (33.3)	5 (14.3)	0.130
Recurrent infections, *n* (%)	3 (20.0)	7 (20.0)	1.00
**Purpose of testing**	​	​	​
Differential diagnosis, *n* (%)	5 (33.3)	17 (48.6)	0.320
Diagnostic clarification, *n* (%)	10 (66.7)	18 (51.4)	0.320
**Symptoms**	​	​	​
Periodic fever, *n* (%)	5 (33.3)	19 (54.3)	0.174
Nonperiodic fever, *n* (%)	6 (40.0)	13 (37.1)	0.849
Arthralgia/arthritis, *n* (%)	8 (53.3)	17 (48.6)	0.758
Chest or abdominal pain, *n* (%)	0	14 (40.0)	0.004
Enterocolitis, *n* (%)	1 (6.7)	9 (25.7)	0.244
Skin rash, *n* (%)	6 (40.0)	6 (17.1)	0.076
Headache/meningitis, *n* (%)	0	3 (8.6)	0.548
Hearing loss, *n* (%)	2 (13.3)	0	0.077
**Laboratory data**	​	​	​
Lymphocyte count (µl), mean (SD)	1,104 (616)	1,566 (620)	0.047
Serum IgG levels (mg/dl), median (IQR)	1,163 (748–1,815)	1,069 (787–1,256)	0.266
Autoantibody positivity, *n* (%)	0	5 (14.3)	0.303
**Pre-genetic treatment**	​	​	​
IVIG, *n* (%)	4 (26.7)	8 (22.9)	1.000
Colchicine, *n* (%)	0	16 (45.7)	0.002
Canakinumab, *n* (%)	0	2 (5.7)	1.000
Glucocorticoids, *n* (%)	6 (40.0)	9 (25.7)	0.309
Immunosuppressants, *n* (%)	2 (13.3)	8 (22.9)	0.702
**Clinical classification**	​	​	​
ID	6 (40.0)	11 (31.4)	0.558
AID	5 (33.3)	19 (54.3)	0.174
Non-ID/AID	4 (26.7)	5 (14.3)	0.423

Patients were classified into two groups according to a final genetic diagnosis, established through multidisciplinary evaluation integrating genetic findings with clinical phenotypes. Patients with a final diagnosis were defined as those in whom genetic findings consistent with IEI were identified and clinically interpreted, whereas patients without a final genetic diagnosis were defined as those in whom such integration did not lead to a conclusive diagnosis. Demographic, clinical, and laboratory characteristics were compared between groups. Variables were compared using either the chi-square test or Fisher’s exact test, depending on cell sizes.

### Computational stratification and feature interpretation

**Figure S1.**
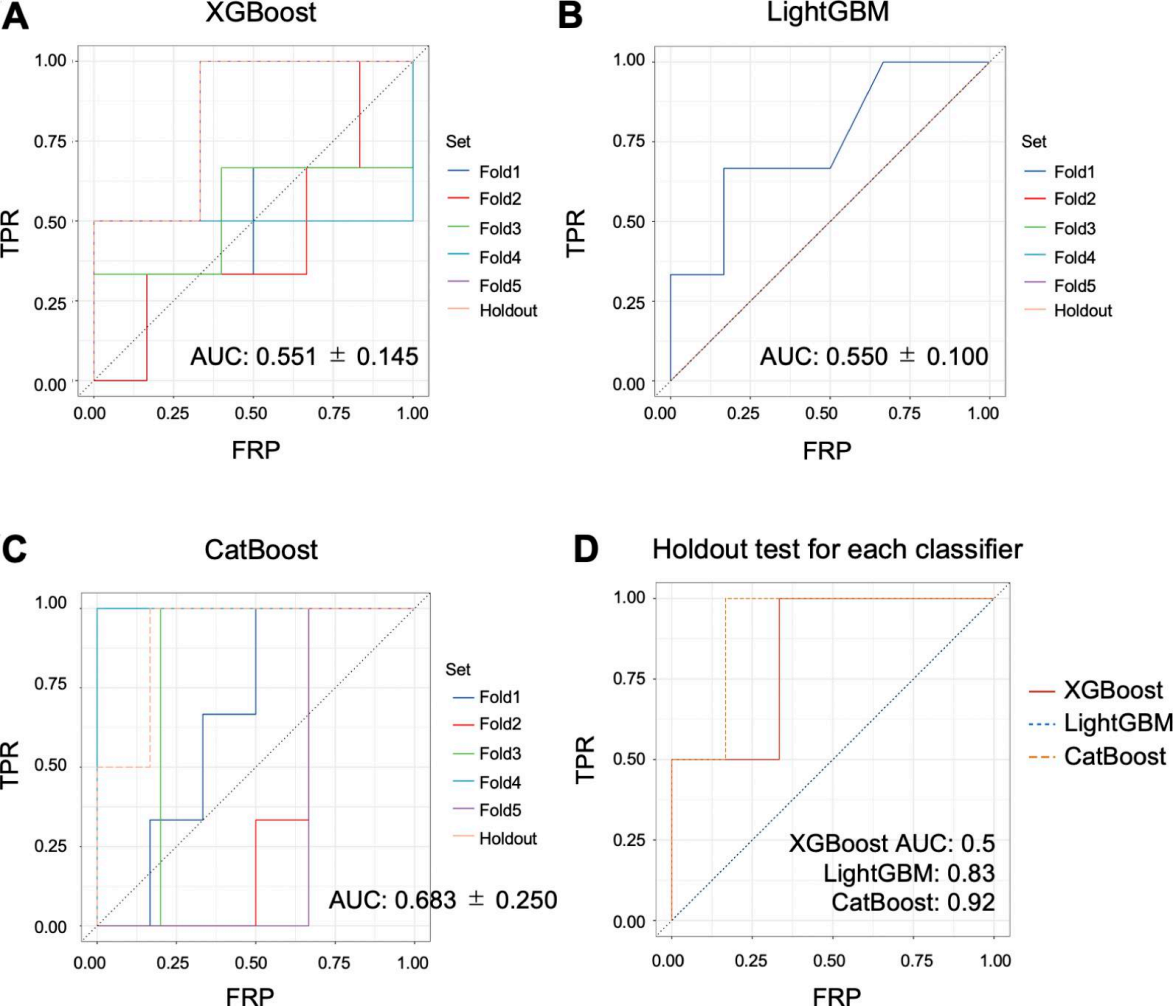
**Predictive performance of gradient boosting classifiers.** Receiver operating characteristic (ROC) curves are shown for XGBoost, LightGBM, and CatBoost models. **(A–C)** Panels depict ROC curves from fivefold cross-validation (colored lines) along with the independent holdout test (dashed line). **(D)** Panel summarizes the ROC curves of the holdout test across the three classifiers. FRP, false positive rate; TPR, true positive rate.

**Figure 2.**
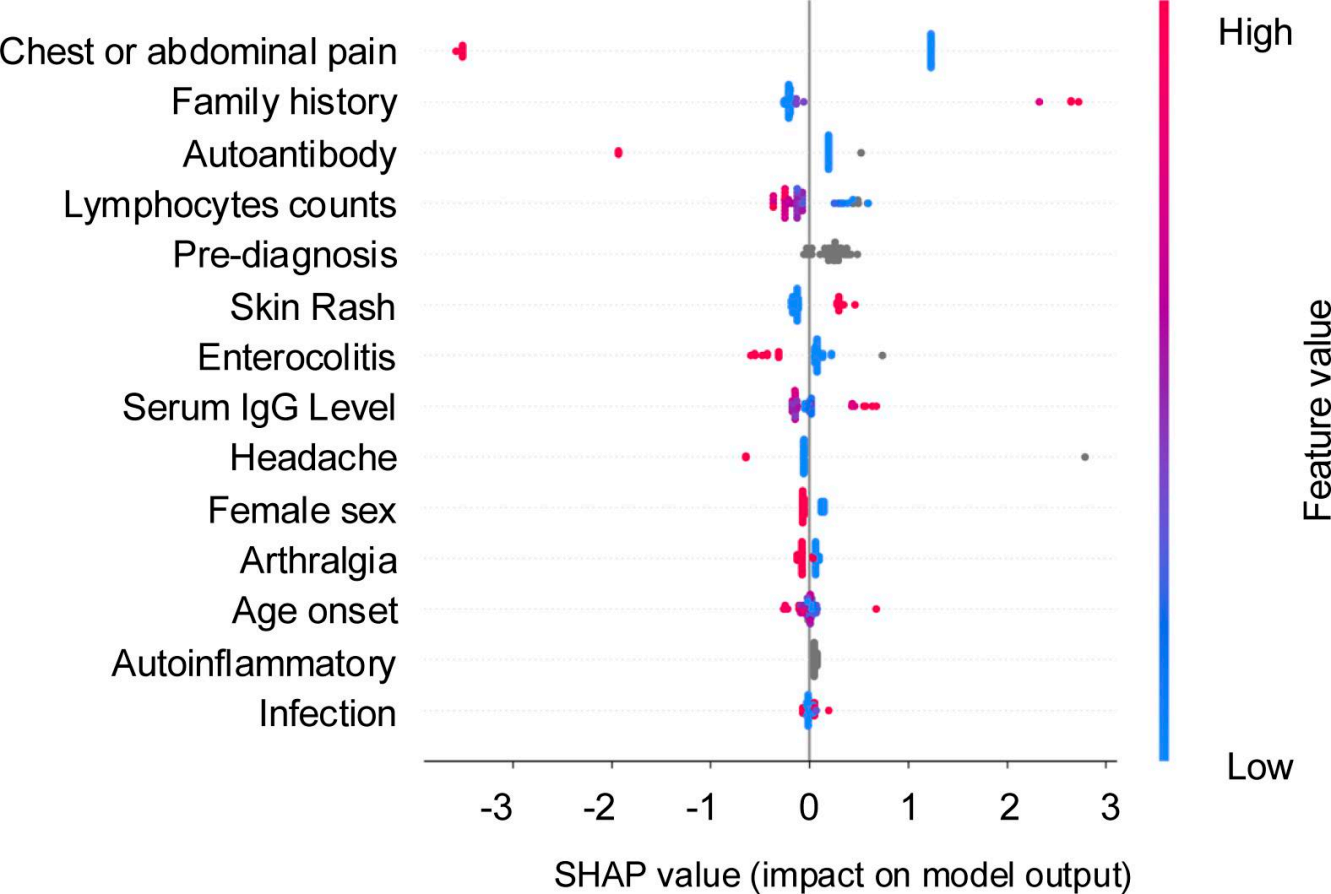
**SHAP-based interpretation of a machine learning classifier for genetic diagnosis in IEI.** SHAP summary plot illustrating feature contributions to CatBoost model output for a final genetic diagnosis consistent with IEI. Each dot represents one patient; color indicates feature value (red = high, blue = low). Positive SHAP values indicate increased contribution to model outputs predicting a final genetic diagnosis, whereas negative values indicate reduced contribution. Family history and lower lymphocyte counts showed relatively positive contributions, while chest or abdominal pain tended to contribute negatively.

**Figure S2.**
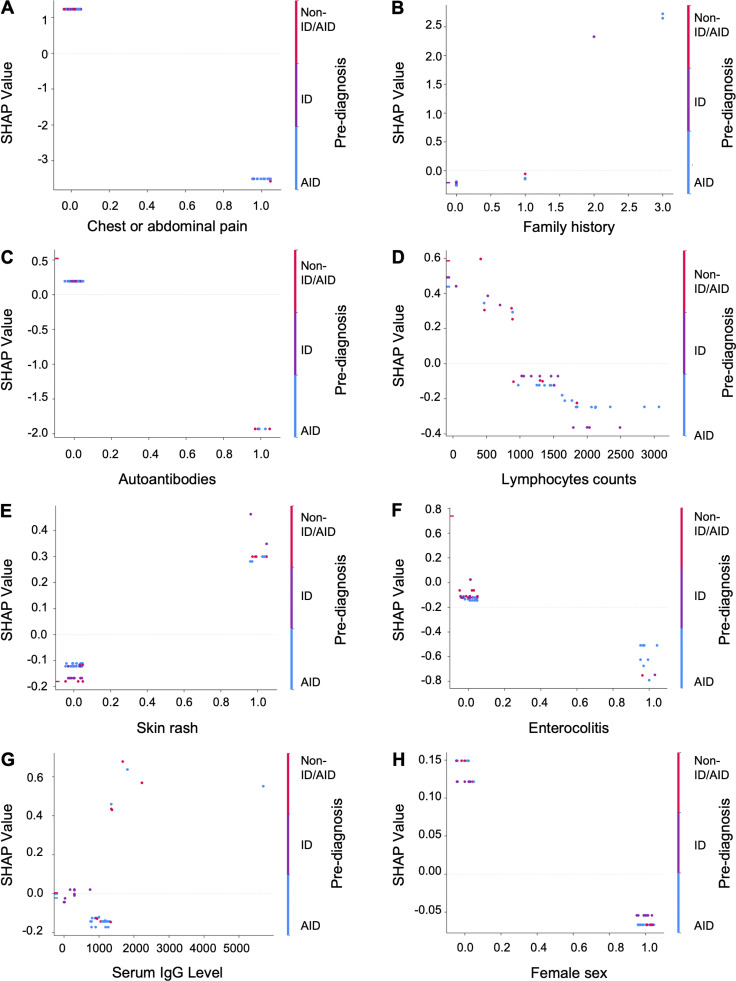
**SHAP dependence analysis in relation to a final genetic diagnosis.** Panels show SHAP dependence plots for variables contributing to the model outputs of a final genetic diagnosis. Each dot represents an individual patient; the x-axis indicates the feature value (binary features are coded as 0/1, where applicable), and the y-axis indicates the SHAP value (positive values increase, and negative values decrease, the predicted probability of a final genetic diagnosis). The dashed horizontal line denotes SHAP = 0. Point colors represent the pre-test clinical classification (AID, ID, and non-ID/AID) as indicated on the color bar. **(A)** Chest/abdominal pain. The presence of chest/abdominal pain was associated with lower SHAP values, indicating reduced contribution to model outputs predicting a final genetic diagnosis across multiple clinical groups. **(B)** Family history. Greater family history scores are associated with progressively increased SHAP values, indicating positive contributions to the model outputs across groups. **(C)** Autoantibodies. Autoantibody positivity showed heterogeneous SHAP contributions without a consistent directional effect across clinical subgroups. **(D)** Lymphocyte counts. Lower lymphocyte counts were associated with higher SHAP values, whereas higher lymphocyte counts contributed negatively to model outputs. **(E)** Skin rash. The presence of skin rash contributed positively to model outputs, particularly among non-ID/AID and AID patients. **(F)** Enterocolitis. Enterocolitis showed variable contribution across patients, with generally modest effects on model prediction. **(G)** Serum IgG. Lower serum IgG levels tended to contribute positively to model outputs, whereas higher IgG levels showed reduced or negative contributions. **(H)** Female sex. Female sex showed relatively small and heterogeneous contributions to model outputs across groups.

Because conventional statistical analyses provided limited insight into the clinical backgrounds associated with a final genetic diagnosis, we further applied machine learning approaches as exploratory analyses to investigate clinical feature patterns and potential nonlinear relationships with genetic diagnosis in adult patients with RMD and suspected IEI. We first trained three gradient boosting classifiers (LightGBM, XGBoost, and CatBoost) on demographic, laboratory, and clinical features. Model performance was assessed by receiver operating characteristic–area under the curve (ROC-AUC), sensitivity, specificity, and accuracy. Among the three models, CatBoost showed the highest mean ROC-AUC performance, although variability across cross-validation folds was substantial (0.638 ± 0.250), as shown in Fig. S1. We then applied SHapley Additive exPlanations (SHAP) analysis to interpret the CatBoost classifier. SHAP summary plots revealed that chest or abdominal pain, family history of the same disease, autoantibody positivity, and lymphocyte counts were among the features contributing to model outputs (Fig. 2). SHAP dependence analysis further demonstrated that the presence of chest or abdominal pain was associated with lower SHAP values, indicating a reduced contribution to model outputs predicting a final genetic diagnosis (Fig. S2). For family history, higher scores were associated with increasing positive SHAP values, showing consistent directional effects across subgroups, although the magnitude of contribution tended to be greater among patients pre-classified as AID (Fig. S2). In contrast, autoantibody positivity demonstrated heterogeneous SHAP contributions across groups without a consistent directional shift, suggesting that its clinical relevance may depend on interactions with other clinical features rather than functioning as a strong discriminator (Fig. S2).

**Figure S3.**
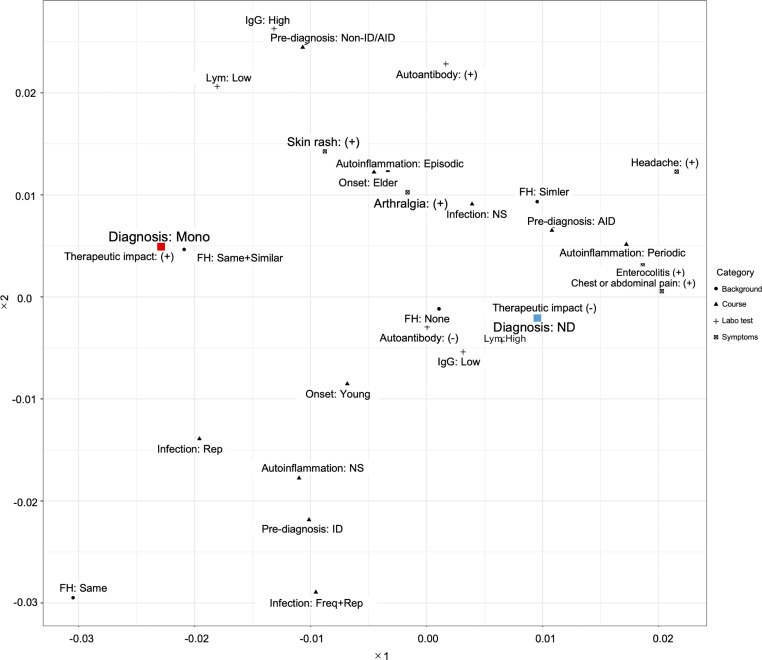
**MCA of clinical features in adult patients with suspected IEI.** MCA biplot of categorical variables included in machine learning preprocessing. Features are grouped by category: background (black circles), clinical course/pre-genetic classification (black triangles), laboratory tests (black crosses), and symptoms (open squares). Labels indicate the variable and its categorical level, e.g., IgG: low or FH: same. Age at onset was categorized as young (<40 years) and elder (≥40 years). Serum IgG levels were classified as low (<1,350 mg/dl) and high (≥1,350 mg/dl). Lymphocyte counts were categorized as low (<900/μl) and high (≥900/μl). Patients with pathogenic/likely pathogenic variants consistent with IEI are indicated by diagnostic labels such as “diagnosis: Mono. ”Genetically undiagnosed patients are marked as “diagnosis: ND.” The two axes (X1 and X2) represent the first and second dimensions, respectively. ND, non-diagnostic; FH, family history; Lym, lymphocyte count; IgG, immunoglobulin G; Rep, repeated; NS, nonspecified; Therapeutic impact (±), treatment modification considered beneficial or not.

**Figure S4.**
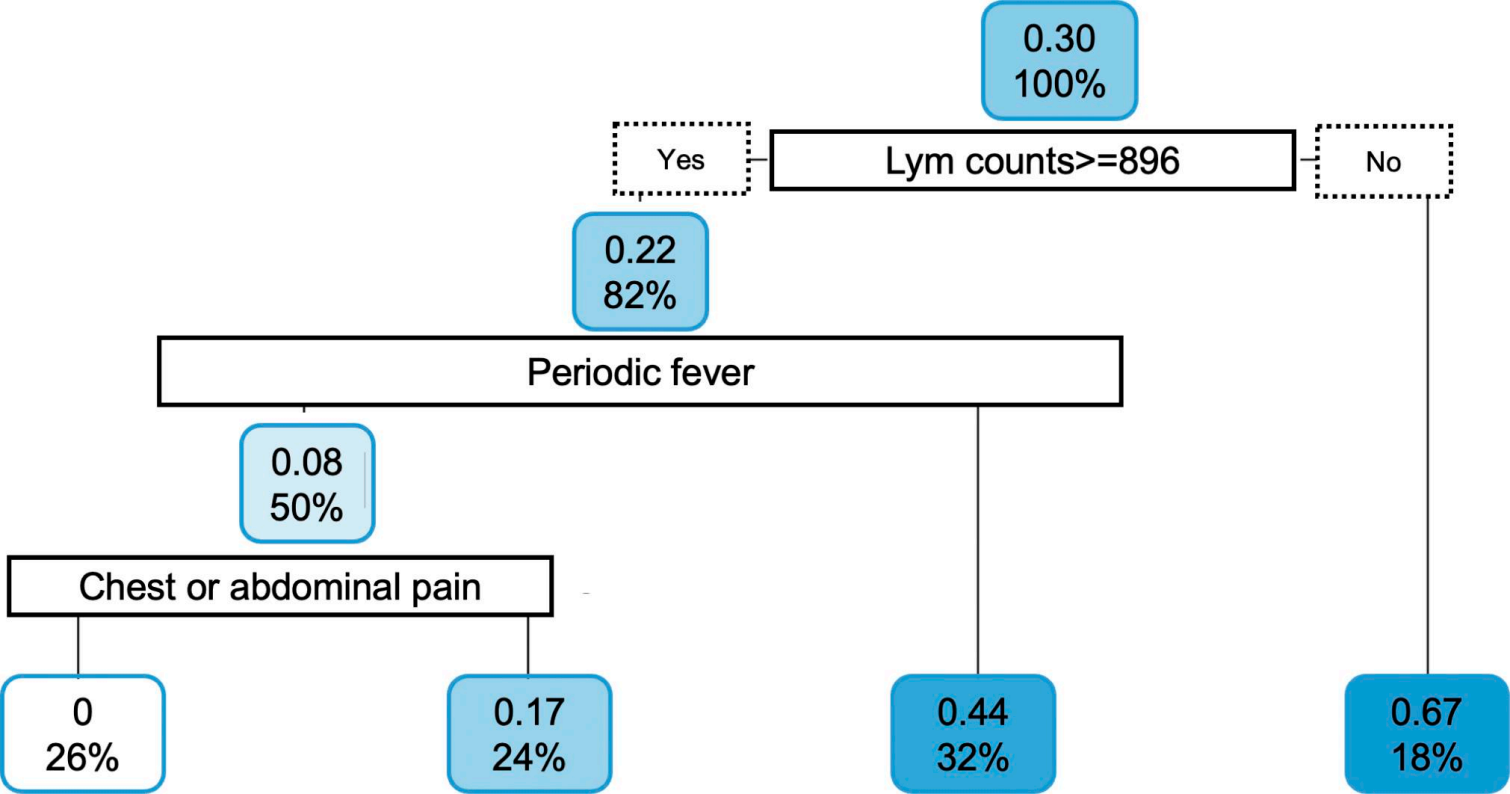
**Decision tree model for clinical stratification.** A CART was constructed using lymphocyte counts, periodic fever, and chest or abdominal pain as input variables. The values within each node represent the predicted probability of harboring a genetic diagnosis (top) and the proportion of patients classified into that node (bottom). Splitting criteria are shown along the branches. This exploratory model provides a clinically interpretable framework, illustrating how combinations of laboratory and clinical features can stratify the likelihood of underlying monogenic IEI in adults with RMD.

To visualize phenotypic overlap across the cohort, multiple correspondence analysis (MCA) was performed using dichotomized categorical variables. The MCA plot demonstrated considerable overlap between patients with and without a final genetic diagnosis, suggesting the difficulty of distinguishing these populations clinically. However, patients with a confirmed final genetic diagnosis showed relative proximity to family history (Fig. S3). Notably, genetically diagnosed patients were also located near cases in which treatment modification was implemented based on the diagnostic findings, whereas those without a genetic diagnosis were positioned closer to cases in which no treatment modification was made. This suggests that genetic diagnosis was closely associated not only with clinical stratification but also with subsequent changes in clinical management. To provide a clinically interpretable framework, we constructed a decision tree (classification and regression tree [CART] model) based on the same feature set. The resulting flowchart suggested a stepwise triage stratification structure with the initial split determined by lymphocyte counts and periodic fever, followed by branching based on chest or abdominal pain (Fig. S4). This rule-based approach illustrates how machine learning–derived feature patterns may support clinical stratification in diagnostically challenging adult patients.

## Discussion

This study investigated the clinical utility of multidisciplinary genomic evaluation in adult patients with suspected IEI who presented with RMD features—a clinical context in which the utility of genetic testing has not been well established. By focusing on these subsets, the study provides novel insights into the clinical application of genomic diagnostics in adult-onset IEI.

Given the considerable heterogeneity of clinical presentations and disease courses, IEIs are challenging to assess at the cohort level. Stratifying patients based on phenotypic presentation has been shown to improve diagnostic accuracy in previous studies (32).

In this study, we classified patients into three subgroups—ID, AID, and an atypical phenotype group (non-ID/AID). The ID group commonly lacked overt physical abnormalities but frequently presented with recurrent or opportunistic infections. The AID group exhibited features consistent with systemic autoinflammatory syndromes, such as arthralgia, serositis, and sensorineural hearing loss (33, 34).

The non-ID/AID group posed a notable diagnostic challenge due to its heterogeneous presentations, which often mimicked RMD without fulfilling the criteria for ID or AID. Many exhibited symptoms such as nonperiodic fever, arthralgia, and skin rash, and some tested positive for autoantibodies, complicating the distinction from classic RMD. Nevertheless, integrated genomic evaluation identified several adult-onset IEI in this group, including VEXAS syndrome (*UBA1* variants) and CTLA4 haploinsufficiency, leading to disease reclassification according to the 2024 IUIS phenotypic framework (9, 35). These findings suggest that phenotype-based clinical classification alone may not fully reflect the underlying molecular pathology in adult-onset IEI. Importantly, several patients in the non-ID/AID group had already received empirical immunosuppressive therapy before genetic testing, underscoring the limitation of phenotype-driven management strategies in diagnostically complex adult patients.

Using pathogenic or likely pathogenic variants interpreted in the context of clinical phenotypes, we identified a final genetic diagnosis consistent with IEI in 30.0% of all patients, which was comparable to the diagnostic yield reported in previous adult IEI cohorts (25–30%) (31, 36). Although the number of genetically diagnosed patients within the non-ID/AID group was small, the relatively high diagnostic yield in this group (44.4%) may reflect the selective enrichment of diagnostically challenging adult patients in whom routine clinical assessment failed to establish a conclusive diagnosis. These patients subsequently underwent multidisciplinary evaluation and advanced genetic testing tailored to the clinical context, including in-house targeted sequencing, the Primary Immunodeficiency Database in Japan (PIDJ)-400 expanded panels, and whole-exome sequencing (WES) (7, 12, 37). For WES performed through the Initiative on Rare and Undiagnosed Diseases (IRUD) framework, variant interpretation undergoes additional review by an external expert panel composed of clinical geneticists and genome specialists who are independent from the primary clinical team, ensuring standardized American College of Medical Genetics and Genomics and the Association for Molecular Pathology (ACMG/AMP)–informed classification and improving diagnostic reliability (38, 39). In selected IRUD framework cases, trio-based analysis clarified inheritance patterns and contributed to variant interpretation, highlighting an additional benefit of family based evaluation even when relatives were not included as independent cases (40, 41). These approaches supported more reliable variant interpretation and clinical integration, suggesting that molecular diagnosis based on ACMG/AMP classification should be interpreted in the context of clinical phenotype, disease course, and treatment response.

In contrast, 35 patients (70.0%) remained without a final genetic diagnosis despite multidisciplinary evaluation integrating genetic findings with clinical phenotypes, possibly due to phenotypic variability, genetic heterogeneity, variants of uncertain significance or the presence of acquired phenocopies of IEI (42). The limitation of current sequencing technologies and incomplete gene coverage may also have played a role. Reanalysis of sequencing data, expansion of gene panels, and incorporation of functional studies may be required to elucidate etiology in these cases (7, 31, 36). Following a final genetic diagnosis, genotype-driven treatment modifications were identified in a subset of patients. In patients with ID, immunoglobulin replacement therapy was initiated following molecular confirmation, whereas in AID targeted therapies such as canakinumab were introduced based on underlying genetic findings. These findings highlight the potential clinical impact of genetic testing in guiding disease-specific therapeutic strategies. In contrast, for many adults with IEIs, including Blau syndrome (NOD2) and VEXAS syndrome (UBA1), therapeutic decisions remain primarily phenotype-driven because genotype-specific therapies are not yet established (43).

Despite diagnostic and therapeutic interventions, two patients died. Both had severe phenotypes associated with *GATA2* deficiencies or *UBA1* variants. In the previously reported GATA2 case, delayed recognition resulted in immunosuppressive treatment initiated as symptomatic management, subsequent severe and refractory opportunistic infections, ultimately precluding curative hematopoietic stem cell transplantation (20). These cases highlight that timely diagnosis is essential to prevent irreversible clinical deterioration (44, 45).

In conventional comparisons of patients with and without a final diagnosis, the presence of a family history of the same disease, chest or abdominal pain, and lymphocyte counts were associated with a final genetic diagnosis. To further explore potential nonlinear relationships among clinical features, we applied machine learning approaches. Because the cohort was preselected, these analyses are exploratory and highlight cohort-specific patterns rather than generalizable diagnostic indicators. The models visualized implicit clinical reasoning and identified chest or abdominal pain and family history as the features contributing to model outputs. These exploratory analyses should be interpreted cautiously because of the limited sample size and potential model instability. Taken together, they support the potential value of multidisciplinary evaluation in diagnostically complex adult cases and illustrate how genetic testing can meaningfully support diagnostic and therapeutic decision-making.

For patients who remain without a final diagnosis despite these efforts, continuous clinical follow-up and comprehensive immunological assessment should be considered to uncover hidden etiologies. Future studies should integrate additional biomarkers, such as cytokine profiles and molecular markers of lymphocyte neogenesis, including T cell receptor excision circles and κ-deleting recombination excision circles, and expand training datasets to improve model robustness and generalizability (46, 47, 48).

This study has several limitations. As a single-center retrospective analysis, potential selection bias influenced the distribution of IEI, and the diagnostic yield may be inflated because only patients undergoing genetic testing were included. Furthermore, the cohort was enriched for patients already considered highly suspicious for IEI based on prior clinical assessment and multidisciplinary evaluation, which may limit the generalizability of the findings. In addition, the inclusion of clinically recognizable classical autoinflammatory diseases—many of whom had already been clinically identified and treated prior to testing—may have led to an overestimation of the overall diagnostic yield. Because the diagnostic difficulty of classical autoinflammatory diseases differs substantially from that of other IEI, future studies should evaluate these groups separately. Moreover, in some cases, exact variant-level information was unavailable because of incomplete historical medical records during long-term follow-up. Finally, the relatively small sample size limited statistical power and may have contributed to model instability in exploratory machine learning analyses.

In conclusion, this study demonstrates that structured clinical classification combined with multidisciplinary genomic evaluation enables the identification of IEI in adult patients with RMD. Genetic testing not only facilitates precise diagnosis but also informs treatment in diagnostically challenging cases. These findings support consideration of genomic evaluation in adult rheumatology practices, especially in atypical or treatment-refractory disease presentations.

## Materials and methods

### Study design and patients

We conducted a retrospective observational study using data extracted from the clinical data warehouse of the Institute of Science Tokyo. Eligible patients were adults (≥18 years) who visited the Department of Rheumatology between March 2018 and September 2024 and were clinically suspected of having IEI. Clinical suspicion was based on the physician’s assessment, considering features such as recurrent infections, an atypical combination of symptoms, chronic inflammation, and/or a family history suggestive of immune dysregulation. Only patients who underwent genetic testing were included in the analysis. Incomplete data about genetic testing―defined as cases in which the performance of testing was unclear from the medical records, results were not documented in sufficient detail, or a final report was unavailable―were excluded from the study. The cohort included individuals referred from other adult departments, as well as patients who had previously undergone genetic testing in pediatric departments (either at our institution or externally) and were subsequently referred for evaluation by rheumatologists. To analyze patients with a final genetic diagnosis consistent with IEI, we excluded patients with familial Mediterranean fever carrying monoallelic variants of *MEFV* and those whose identified variants did not fulfill the ACMG/AMP criteria for pathogenic or likely pathogenic classification from downstream analysis (5, 49). A diagnostic flowchart summarizing the inclusion process and clinical classification framework is provided in Fig. 1.

### Genetic testing workflow and analytical procedure

#### Patient selection and clinical workflow for genetic testing

**Figure S5.**
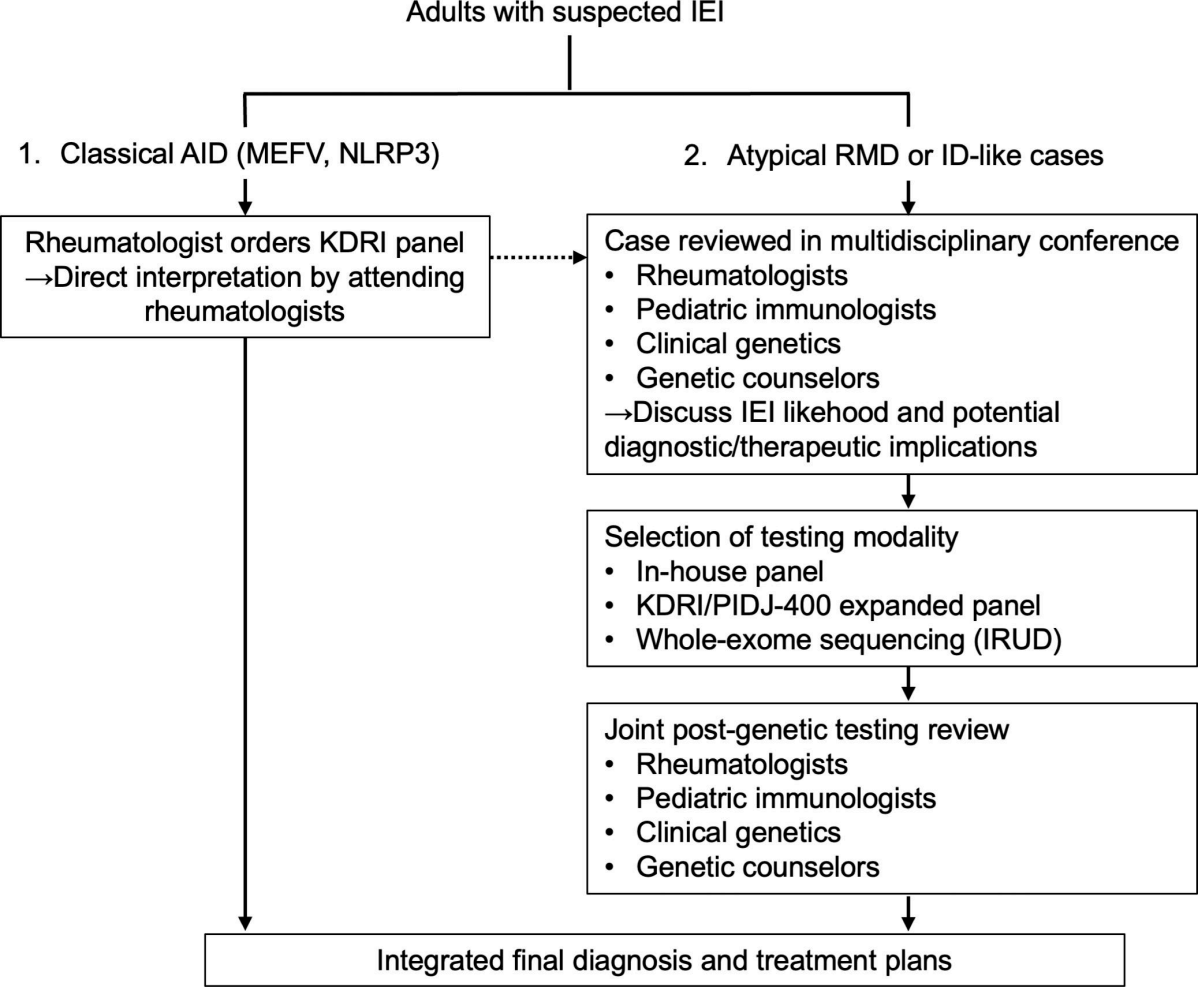
**Clinical workflow for multidisciplinary genomic evaluation in adults with suspected IEI.** Patients with clinically apparent classical autoinflammatory diseases (e.g., *MEFV*- or *NLRP3*-associated disorders) were initially evaluated by rheumatologists, who ordered targeted testing through the KDRI and directly interpreted the results when the phenotype was concordant. In contrast, patients with atypical rheumatic manifestations or ID-like features were systematically reviewed in multidisciplinary conferences involving rheumatologists, pediatric immunologists, clinical geneticists, and genetic counselors. These discussions addressed IEI likelihood and the potential diagnostic or therapeutic implications of genetic findings. Based on this review, the appropriate testing modality was selected—ranging from in-house targeted panels to KDRI/PIDJ-400 expanded panels or WES through the Initiative on IRUD program. All pathogenic or likely pathogenic variants identified through genetic testing were subsequently interpreted jointly across specialties, and final clinical interpretation was performed in the context of clinical phenotypes and disease course.

A structured, multidisciplinary workflow was used to determine whether patients should proceed to genetic testing (Fig. S5). Patients with clinical features suggestive of classical monogenic autoinflammatory disease―such as *MEFV*- or *NLRP3*-associated disorders―were initially evaluated by rheumatologists, who ordered targeted genetic testing through the Kazusa DNA Research Institute (KDRI). When the clinical presentation was highly concordant with the suspected diagnosis, treatment decisions were made directly by the attending rheumatologist based on these results. However, if discrepancies between the clinical phenotype and the suspected diagnosis were noted, or if ID-like manifestations or recurrent infections were present, these classical autoinflammatory disease cases were also presented at a departmental multidisciplinary conference.

Patients with atypical inflammatory phenotypes, ID-like features, or symptoms insufficiently explained by conventional RMD were routinely reviewed at multidisciplinary conferences involving rheumatologists, pediatric immunologists, clinical geneticists, and genetic counselors. During these conferences, the likelihood of IEI, the potential therapeutic implications of the genetic findings, and the appropriate genetic testing modality were discussed.

#### Genetic testing platforms

Genetic analyses were performed using four platforms: (1) an in-house targeted panel, (2) targeted panels from KDRI, (3) the PIDJ-400 expanded panels, and (4) WES through the IRUD framework. Gene panels from KDRI were designed to cover well-established genes associated with ID and autoinflammatory diseases (Table S6). For broader coverage, we applied an expanded targeted sequencing panel covering ∼400 genes associated with IEI. This panel was developed under the PIDJ framework, based on the 2019 classification of IEI genes by the IUIS (50, 51). WES was performed within the IRUD framework, a nationwide diagnostic initiative in Japan. Trio-based sequencing comprising the proband and both biological parents is routinely implemented whenever feasible, enabling assessment of de novo variants, segregation, and inheritance patterns. As part of this framework, variant interpretation included an additional review by an external expert panel composed of clinical geneticists and genome specialists (38, 39). A complete flowchart of the testing workflow and selection logic is presented in Fig. S5.

#### Selection of testing modality

Based on multidisciplinary evaluations, patients were directed to at least one of the available platforms: an in-house panel, KDRI panels, the PIDJ-400 expanded panel, or WES through IRUD. The PIDJ-400 panel and WES through IRUD were preferentially selected for cases in which broader differential diagnoses were considered or when initial targeted KDRI testing was inconclusive.

#### Variant interpretation and diagnostic integration

Variants were interpreted in accordance with guidelines of ACMG/AMP, using population allele frequency, Combined Annotation Dependent Depletion scores, and other supportive evidence (49). Variants classified as pathogenic or likely pathogenic according to ACMG/AMP guidelines were jointly reviewed by a multidisciplinary team comprising rheumatologists, pediatric immunologists, clinical geneticists, and genetic counselors. Final clinical interpretation was performed by integrating these molecular findings with phenotypes, disease course, and treatment response. In this study, all genetic diagnoses were subsequently categorized according to the 2024 IUIS phenotypic classification for human IEI (52).

### Clinical classification of patients

Most of the patients in this study had several symptoms suggestive of RMD, such as musculoskeletal complaints, unexplained fever, or skin manifestations. Patients were classified into three groups based on their clinical presentation and treatment history:

1. ID: Patients with recurrent, persistent, or opportunistic infections and/or requiring regular immunoglobulin replacement therapy.
2. AID: Patients without clear features of ID but with periodic inflammatory episodes, including fever, chest or abdominal pain, skin rash, or sensorineural hearing loss.
3. Non-ID/AID: Patients who did not meet criteria for either ID or AID. This group included cases initially diagnosed with RMD such as undifferentiated connective tissue disease or seronegative arthritis. These patients exhibited atypical or overlapping features (e.g., nonperiodic fever, nonspecific skin rash, or negative or atypical autoantibodies) and unusual disease progression or poor response to conventional immunosuppressive/antirheumatic treatments.

### Statistical analysis

Univariate analyses were performed to compare patients with and without pathogenic or likely pathogenic variants consistent with IEI. Categorical variables were compared using the chi-square test or Fisher’s exact test, as appropriate, and continuous variables were compared using the Mann–Whitney U test. Clinical data were aggregated and summarized using SPSS version 30.

### Machine learning pipeline

We developed exploratory machine learning models to investigate clinical patterns associated with a genetic diagnosis consistent with IEI from our cohort data. Input variables consisted of demographic data, laboratory values (e.g., serum IgG levels and lymphocyte count), and clinical features (e.g., skin rash, autoantibody positivity, and family history). Prior to model training, feature engineering was performed (Table S7). For patients who were already receiving immunoglobulin supplementation therapy, serum IgG levels were coerced to 300 mg/dl. Missing IgG values were also imputed as 300 mg/dl. This threshold was chosen to approximate clinically relevant hypogammaglobulinemia as described in previous studies linking IgG < 400 mg/dl to PID (19, 53, 54). Family history was categorized into four ordered classes (same and similar, same only, similar only, or none), and history of infections was similarly categorized (frequent and recurrent, frequent only, recurrent only, or none). These ordinal categories were converted to integers to preserve ordinal relationships. Missing values were otherwise imputed using the median (for continuous variables) or the mode (for categorical variables).

#### Classification model performance

Three gradient boosting implementations—LightGBM (v4.6.0), XGBoost (v3.2.0), and CatBoost (v1.2.8)—were trained on the dataset (55, 56). Model performance was evaluated using a nested cross-validation with a 15% independent holdout set. Fivefold outer cross-validation loops were used for model evaluation, with fivefold inner loops for hyperparameter optimization via Optuna (v4.7.0). Final models were trained on 90% of the data and tested on the holdout set. Discrimination was assessed by ROC-AUC as the primary metric, alongside sensitivity, specificity, and accuracy.

#### Model interpretation using SHAP analysis

To enhance the interpretability of the machine learning model, we applied SHAP to the best-performing gradient boosting classifier (57). SHAP values were computed for each input variable to quantify its marginal contribution to the predicted probability of IEI. Summary plots were generated to visualize the overall impact, direction, and importance of each feature across the cohort.

#### Exploratory analysis: MCA

To explore latent structures in categorical data, MCA was performed using features such as autoantibody positivity, skin rash, and family history (58). Patients with complete data (*n* = 50) were included. The results were projected in two dimensions to assess clustering patterns between genetically diagnosed and undiagnosed groups. MCA was performed in R (v4.5.2) using the MASS package (v7.3-65).

#### Clinically interpretable classification: Decision tree analysis

To develop a rule-based exploratory classification framework, we constructed a CART model using the same feature set (59). The Gini impurity criterion was used for node splitting, and tree depth was restricted to reduce overfitting. The resulting tree provided a stepwise, clinically interpretable algorithm for triage based on key variables.

All statistical and machine learning analyses were performed using Python (version 3.12.12) with XGBoost, LightGBM, CatBoost, SHAP, and prince libraries. The CART model was implemented in R (v4.5.2) using the rpart package (v4.1.24).

### Online supplemental material

Supplementary information includes additional details regarding testing panels, preprocessing strategies, clinical characteristics, and supplementary analyses. Fig. S1 shows predictive performance of gradient boosting classifiers. Fig. S2 shows SHAP dependence analysis in relation to a final genetic diagnosis. Fig. S3 shows MCA of clinical features in adult patients with suspected IEI. Fig. S4 shows decision tree model for clinical stratification. Fig. S5 shows clinical workflow for multidisciplinary genomic evaluation in adults with suspected IEI. Table S1 shows presumed diagnoses assigned by primary physicians in the non-ID/AID group. Table S2 shows detailed infection history and prophylactic treatments in patients with the ID group. Table S3 shows serum immunoglobulin levels in patients receiving immunoglobulin replacement therapy at the time of genetic testing. Table S4 shows genetic variants identified in patients with a final genetic diagnosis. Table S5 shows a summary of fatal cases identified in the cohort. Table S6 shows diagnostic gene panels provided by KDRI. Table S7 shows variable preprocessing and encoding strategy for MCA and machine learning analyses.

## Ethics approval

This study was performed in line with the principles of the Declaration of Helsinki. This study was approved by the Institutional Review Board of the Institute of Science Tokyo (Approval No.: M2024-032). Informed consent was obtained using an opt-out approach, in accordance with institutional ethical guidelines for retrospective studies involving anonymized patient data. For patients undergoing genetic testing at the Institute of Science Tokyo, written informed consent was obtained individually. This included specific consent for the reporting of incidental genomic findings in accordance with ACMG guidelines. In addition, genetic testing conducted under the PIDJ-related research was separately approved by the Institutional Review Board of the Institute of Science Tokyo (Approval No.: G2019-004), and written informed consent was likewise obtained individually from each participant.

## Supplementary Material

Table S1 shows presumed diagnoses assigned by primary physicians in the non-ID/AID group.

Table S2 shows detailed infection history and prophylactic treatments in patients with the ID group.

Table S3 shows serum immunoglobulin levels in patients receiving immunoglobulin replacement therapy at the time of genetic testing.

Table S4 shows genetic variants identified in patients with a final genetic diagnosis.

Table S5 shows summary of fatal cases identified in the cohort.

Table S6 shows diagnostic gene panels provided by KDRI.

Table S7 shows variable preprocessing and encoding strategy for MCA and machine learning analyses.

## Data Availability

The novel disease-causing genetic variants identified in this study have been deposited in the ClinVar database under the following submission numbers: SCV007520436, SCV007520438, and SCV007612062.1. All data supporting the findings of this study are included within the article and its supplementary materials. Additional information is available from the corresponding author upon reasonable request. Individual-level WES data generated through the IRUD framework are not publicly available due to restrictions related to participant privacy, informed consent, and ethical approval for human genomic data.
