# Supplementary material for: Multidisciplinary genomic evaluation reveals adult inborn errors of immunity with rheumatic features
Source: J Hum Immun. 2026 Aug 10;2(5):e20250218. doi: 10.70962/jhi.20250218 (PMC13455650; doi:10.70962/jhi.20250218)
Supplement: Supplementary file 1 [file jhi_20250218_tables1.docx]

**Table S1. Presumed diagnoses assigned by primary physicians in the Non-ID/AID group**

| **Disease** | n |
| --- | --- |
| Systemic lupus erythematosus | 1 |
| Atypical familial Mediterranean fever | 1 |
| Dermatomyositis | 1 |
| Incomplete Behçet’s disease | 1 |
| CTLA4 haploinsufficiency | 1 |
| Articular sarcoidosis | 1 |
| Sweet's syndrome | 1 |
| Relapsing polychondritis | 1 |
| Autoinflammatory disease | 1 |

This table summarizes the presumed clinical diagnoses assigned by attending physicians prior to genetic testing in patients classified under the “Non-ID/AID” category. These diagnoses were based on clinical features and used to guide further genetic evaluation. Some of these conditions later overlapped with monogenic immune disorders.
