# Supplementary material for: Multidisciplinary genomic evaluation reveals adult inborn errors of immunity with rheumatic features
Source: J Hum Immun. 2026 Aug 10;2(5):e20250218. doi: 10.70962/jhi.20250218 (PMC13455650; doi:10.70962/jhi.20250218)
Supplement: Supplementary file 2 [file jhi_20250218_tables2.docx]

**Table S2. Detailed infection history and prophylactic treatments in patients with the immunodeficiency group**

|  | Frequent infections | Opportunistic infections | Fungal infections | Upper respiratory tract infections | Other infections or complications | Prophylaxis (TMP-SMX) | Prophylaxis (Itraconazole) |
| --- | --- | --- | --- | --- | --- | --- | --- |
| 1 | No | Yes | No | No | EBV viremia | Yes | No |
| 2 | Yes | No | No | Yes | Suspected pneumonia | No | No |
| 3 | No | No | No | No | Chronic prostatitis | No | No |
| 4 | Yes | Yes | No | No | Recurrent pneumonia | Yes | Yes |
| 5 | No | No | No | Yes | Sinusitis | Yes | No |
| 6 | Yes | No | No | No | None | No | No |
| 7 | No | Yes | No | No | None | No | No |
| 8 | No | No | Yes | Yes | None | No | Yes |
| 9 | Yes | No | No | No | Pneumonia | No | No |
| 10 | Yes | No | No | No | Pneumonia | No | No |
| 11 | No | No | No | No | None | No | No |
| 12 | Yes | No | No | No | Pneumonia | No | No |
| 13 | Yes | No | No | Yes | None | No | No |
| 14 | Yes | No | No | Yes | Otitis media | No | No |
| 15 | Yes | Yes | Yes | No | Sinusitis, pneumonia, candidemia, CMV, Gastric ulcer | Yes | Yes |
| 16 | Yes | No | No | Yes | None | No | No |
| 17 | Yes | Yes | No | Yes | Herpes zoster, otitis media | No | No |

This table summarizes infection histories and prophylactic treatments in patients classified into the immunodeficiency (ID) group. Frequent infections were defined as recurrent clinically significant infections requiring repeated medical attention. Upper respiratory tract infections include recurrent sinusitis, otitis media, or bronchitis. Opportunistic and fungal infections were classified based on clinical diagnoses; in this study, “opportunistic infections” were defined broadly to include infections associated with impaired immune function, such as EBV viremia, herpes zoster, and cytomegalovirus infection, rather than being restricted to strictly defined opportunistic pathogens. Prophylactic treatments included trimethoprim–sulfamethoxazole (TMP–SMX) and itraconazole. Additional clinically relevant information is provided in the column.
