# Supplementary material for: Multidisciplinary genomic evaluation reveals adult inborn errors of immunity with rheumatic features
Source: J Hum Immun. 2026 Aug 10;2(5):e20250218. doi: 10.70962/jhi.20250218 (PMC13455650; doi:10.70962/jhi.20250218)
Supplement: Supplementary file 3 [file jhi_20250218_tables3.docx]

**Table S3. Serum immunoglobulin levels in patients receiving immunoglobulin replacement therapy at the time of genetic testing**

|  | Pre-genetic testing classification | IgG (mg/dL) | IgA (mg/dL) | IgM (mg/dL) |
| --- | --- | --- | --- | --- |
| 1 | ID | 4513 | 121 | 226 |
| 2 | ID | 593 | 179 | 96 |
| 3 | ID | 484 | 353 | 115 |
| 4 | ID | 454 | 34 | 31 |
| 5 | ID | 1172 | 214 | 308 |
| 6 | ID | 262 | 16 | 13 |
| 7 | ID | 10 | LOD | 1 |
| 8 | ID | 411 | 48 | 93 |
| 9 | ID | 17 | 1 | 9 |
| 10 | ID | 661 | 36 | 49 |
| 11 | ID | LOD | LOD | LOD |
| 12 | ID | 956 | 8 | 8 |

Serum immunoglobulin G (IgG), immunoglobulin A (IgA) and immunoglobulin M (IgM) levels were measured at the time of genetic testing in patients receiving immunoglobulin replacement therapy. Case #10 represents measurements obtained after the initiation of immunoglobulin replacement therapy.

LOD indicates values below the lower limit of detection.
