# Supplementary material for: Multidisciplinary genomic evaluation reveals adult inborn errors of immunity with rheumatic features
Source: J Hum Immun. 2026 Aug 10;2(5):e20250218. doi: 10.70962/jhi.20250218 (PMC13455650; doi:10.70962/jhi.20250218)
Supplement: Supplementary file 4 [file jhi_20250218_tables4.docx]

**Table S4. Genetic variants identified in patients with a final genetic diagnosis**

| **Patient ID** | **Gene** | **Genetic testing platform** | | | | | **Variants** | | | | | | | **Population frequency** | | |
| --- | --- | --- | --- | --- | --- | --- | --- | --- | --- | --- | --- | --- | --- | --- | --- | --- |
|  |  | **Target sequence** | **DNA panel** | **IUIS >400 genes** | **IRUD** | **family segregation analysis** | **Nucleotide change (c.) (HGVS)** | **Protein change (p.) (HCVS)** | **RefSeq** | **rs ID (dbSNP)** | **Zygosity** | **Inheritance pattern** | **Variant type** | **gnomAD, overall** | **gnomAD, East Asian** | **ToMMo 61KJPN** |
| 1 | *NLRP3* | Yes | No | No | No | No | c.1321G>A | p.Ala441Thr | NM_004895.4 | Not registered | heterozygosity | AD | Unavailable (historical testing; records not retrievable) | Not applicable | Not applicable | 0.E+00 |
| 2 | *NLRP3* | Yes | No | No | No | No | c.1321G>A | p.Ala441Thr | NM_004895.4 | Not registered | heterozygosity | AD | Unavailable (historical testing; records not retrievable) | Not applicable | Not applicable | 0.E+00 |
| 3 | *NLRP3* | No | No | Yes | No | No | c.1322C>T | p.Ala441Val | NM_004895.4 | rs121908146 | heterozygosity | AD | Unavailable (historical testing; records not retrievable) | 1.E-05 | 0.E+00 | 0.E+00 |
| 4 | *NLRP3* | N.D. | N.D. | N.D. | N.D. | N.D. | c.2582A>G | p.Tyr861Cys | NM_004895.4 | Not registered | heterozygosity | AD | Missense | Not applicable | Not applicable | 0.E+00 |
| 5 | *CTLA4* | Yes | No | No | No | No | c.C34T | p.Gln12X | NM_005214.5 | Not registered | heterozygosity | AD | Unavailable (historical testing; records not retrievable) | Not applicable | Not applicable | 0.E+00 |
| 6 | *CTLA4* | No | Yes | No | No | No | c.485delT | p.Phe162fs | NM_005214.5 | Not registered | heterozygosity | AD | Frameshift | Not applicable | Not applicable | Not applicable |
| 7 | *NFKB2* | Yes | No | No | No | No | Unavailable (historical testing; records not retrievable) | p.Arg853X | NM_001322934 | rs397514332 | heterozygosity | AD | Unavailable (historical testing; records not retrievable) | 0.E+00 | 0.E+00 | 0.E+00 |
| 8 | *NFKB1* | No | Yes | No | No | No | c.1883dupA | His628GlnfsTer2 | NM_003998.4 | Not registered | heterozygosity | AD | Frameshift | Not applicable | Not applicable | Not applicable |
| 9 | *UBA1* | Yes | No | No | No | No | c.121A>C | p.Met41Leu | NM_003334.4 | rs1936307795 | Somatic mosaicism | Somatic (X-Linked) | Unavailable (historical testing; records not retrievable) | Not applicable | Not applicable | 0.E+00 |
| 10 | *UBA1* | Yes | No | No | No | No | c122T>C | p.Met41Thr | NM_003334.4 | rs782416867 | Somatic mosaicism | Somatic (X-Linked) | Unavailable (historical testing; records not retrievable) | Not applicable | Not applicable | 0.E+00 |
| 11 | *NOD2* | No | Yes | No | No | No | c.1000C>T | p.Arg334Trp | NM_001370466.1 | Not registered | heterozygosity | AD | Missense | Not applicable | Not applicable | Not applicable |
| 12 | *PRF1* | Yes | Yes | Yes | No | No | c.133G>A | p.Gly45Arg | NM_001083116.3 | rs578092914 | Compound heterozygosity | AR | Missense | 7.E-06 | 0.E+00 | 8.E-03 |
|  |  |  |  |  |  |  | c.1153C>T | p.Arg385Trp | NM_001083116.3 | rs551046401 |  |  |  | 2.E-04 | 0.E+00 | 6.E-04 |
| 13 | *GATA2* | No | Yes | Yes | Yes | Yes | c.1051A>G | p.Asn351Asp | NM_032638.5 | Not registered | heterozygosity | AD | Missense | Not applicable | Not applicable | Not applicable |
| 14 | *ALPK1* | Yes | No | No | No | No | c.710C>T | p.Thr237Met | NM_025144.4 | rs1052954321 | heterozygosity | AD | Unavailable (historical testing; records not retrievable) | 7.E-07 | 0.E+00 | 0.E+00 |
| 15 | *ICOS* | Yes | No | No | No | Yes | c.285delT | p.Phe95fs*121 | NM_012092.4 | Not registered | homozygosity | AR | Frameshift deletion | Not applicable | Not applicable | Not applicable |

Continued

| **Patient ID** | **In silico prediction** | | | | | **ClinVar clinical significance** | **ACMG** | | **Genetic interpretation** | **Pre-test classification** | **Post-test classification** | **Final genetic diagnosis** |
| --- | --- | --- | --- | --- | --- | --- | --- | --- | --- | --- | --- | --- |
|  | **SIFT, Score** | **SIFT, Interpretation** | **CADD, PHRED** | **Polyphen, Score** | **Polyphen, Interpretation** |  | **class** | **Evidence Codes** |  |  |  |  |
| 1 | 0.E+00 | Deleterious | 25.8 | Not applicable | Probably damaging | Pathogenic | Likely Pathogenic | PM2, PM5, PM1 | Disesae-causing | AID | Autoinflammatory diseases | Cryopyrin-Associated Periodic Syndromes |
| 2 | 0.E+00 | Deleterious | 25.8 | Not applicable | Probably damaging | Pathogenic | Likely Pathogenic | PM2, PM5, PM1 | Disesae-causing | AID | Autoinflammatory diseases | Cryopyrin-Associated Periodic Syndromes |
| 3 | 0.E+00 | Deleterious | 24.5 | 1 | Probably damaging | Pathogenic | Pathogenec | PS1, PM2, PP3 | Disesae-causing | AID | Autoinflammatory diseases | Cryopyrin-Associated Periodic Syndromes |
| 4 | 0.E+00 | Deleterious | 25.6 | 1 | Probably damaging | Uncertain significance | Likely Pathogenic | PM2, PM5, PP3 | Disesae-causing | AID | Autoinflammatory diseases | Cryopyrin-Associated Periodic Syndromes |
| 5 | Not applicable | Not applicable | 35.0 | Not applicable | Stop-gain | Not reported | Pathogenec | PVS1, PM2 | Disesae-causing | Non-ID/AID | Diseases of immune dysregulation | CTLA4 Haploinsufficiency |
| 6 | Not applicable | Not applicable | 34.0 | Not applicable | Frameshift | Not reported | Likely Pathogenic | PVS1, PM2 | Disesae-causing | ID | Disease of immune dysregulation | CTLA4 Haploinsufficiency |
| 7 | Not applicable | Not applicable | 35.0 | Not applicable | Stop-gain | Pathogenic | Pathogenic | PVS1, PM2 | Disesae-causing | ID | Predominantly antibody deficiencies | NFKB2 Deficiency |
| 8 | Not applicable | Not applicable | 35.0 | Not applicable | Frameshift | Not reported | Pathogenec | PVS1, PM2 | Disesae-causing | ID | Predominantly antibody deficiencies | NFKB1 Deficiency |
| 9 | 1.E-02 | deleterious - low confidence | 22.8 | 0.022 | Benign | Conflicting classifications of pathogenicity | Not applicable (interpreted based on prior reports of VEXAS syndrome) | PS3, PM2, PP3 | Disesae-causing | Non-ID/AID | Somatic associated autoinflammatory diseases | VEXAS Syndrome |
| 10 | 1.E-02 | deleterious - low confidence | 22.9 | 0.007 | Benign | Conflicting classifications of pathogenicity | Not applicable (interpreted based on prior reports of VEXAS syndrome) | PS3, PM2, PP3 | Disesae-causing | Non-ID/AID | Somatic associated autoinflammatory diseases | VEXAS Syndrome |
| 11 | 0.E+00 | Deleterious | 25.8 | 1 | Probably damaging | Pathogenic | Pathogenic | PM2, PM5, PP3 | Disesae-causing | Non-ID/AID | Autoinflammatory diseases | Blau Syndrome |
| 12 | 0.E+00 | Deleterious | 24.8 | 0.998 | Probably damaging | Pathogenic/Likely pathogenic | Pathogenic | PM3, PS1, PM2, PP3 | Disesae-causing | ID | Diseases of immune dysregulation | Perforin Deficiency |
|  | 0.E+00 | Deleterious | 24.5 | 1 | Probably damaging | Conflicting classifications of pathogenicity | Likely Pathogenic | PM1, PM2, PP2 |  |  |  |  |
| 13 | 0.E+00 | Deleterious | 28.1 | 1 | Probably damaging | Not reported | Likely Pathogenic | PM2, PP3 | Disesae-causing | ID | Combined immune deficiency | GATA2 Deficiency |
| 14 | 5.E-02 | tolerated | 26.8 | 0.952 | Probably damaging | Pathogenic | Pathogenic | PM2 | Disesae-causing | AID | Autoinflammatory diseases | ROSAH syndrome |
| 15 | Not applicable | Not applicable | 35.0 | Not applicable | Frameshift | Not reported | Likely Pathogenic | PVS1, PM2 | Disesae-causing | ID | Predominantly antibody deficiencies | ICOS deficiency |

This table summarizes genetic variants identified in patients in whom a final genetic diagnosis consistent with inborn errors of immunity was established through multidisciplinary evaluation integrating ACMG/AMP-classified variants with clinical phenotypes, disease course and treatment response.

For each patient, the table includes the affected gene, reported nucleotide and protein changes according to HGVS nomenclature with reference transcript information, zygosity, inheritance pattern, variant type, population allele frequency (gnomAD v4.1.0, when available) **as well as population-specific frequencies from the East Asian subset of gnomAD and the ToMMo 61KJPN reference panel when available**, in silico prediction scores **including SIFT and CADD PHRED scores**, and ACMG/AMP classification where applicable**.**

The “Genetic interpretation” column reflects the clinical interpretation adopted in this study and categorized findings as follows: “Disease-causing”, defined as cases with sufficient molecular evidence, including biallelic pathogenic variants in autosomal recessive disorders or pathogenic variants consistent with the expected inheritance pattern.

ACMG/AMP classification represents standardized variant-level assessment and is reported independently from the final clinical genetic interpretation.

Missing data attributable to historical genetic testing for which original reports could not be retrieved—due to institutional transfer, record retention limits, or unavailable archived documentation—were indicated as “Unavailable (historical testing; records not retrievable).”

The term “Not applicable” is used only in specific contexts where standard variant-level annotation was not appropriate, including somatic variants, copy number or structural variants, or historical genetic diagnoses for which raw sequencing data were unavailable.

Variant annotations and supporting information were obtained from publicly available databases, including ClinVar, gnomAD (v4.1.0), Ensembl Genome Browser, and UniProt.
