# Supplementary material for: Multidisciplinary genomic evaluation reveals adult inborn errors of immunity with rheumatic features
Source: J Hum Immun. 2026 Aug 10;2(5):e20250218. doi: 10.70962/jhi.20250218 (PMC13455650; doi:10.70962/jhi.20250218)
Supplement: Supplementary file 5 [file jhi_20250218_tables5.docx]

**Table S5. Summary of fatal cases identified in the cohort**

|  | Age at Testing | Age at Death | Sex | Genetic Classification | Final Diagnosis | Clinical Features |
| --- | --- | --- | --- | --- | --- | --- |
| 1 | 34 | 35 | Female | ID | GATA2 deficiency | Sarcoidosis-like systemic inflammation, Pulmonary alveolar proteinosis, MDS, Recurrent infections |
| 2 | 72 | 73 | Male | Non-ID/AID | VEXAS syndrome | Sweet syndrome, MDS |

This table summarizes the clinical characteristics and causes of death in two patients who died during the study period. The two patients had been classified as immunodeficiency (ID) and non-ID/AID, respectively. Both patients presented with complex disease phenotypes and ultimately died of infectious complications or pulmonary involvement. Case 1 has been previously reported (20).
