# Supplementary material for: Multidisciplinary genomic evaluation reveals adult inborn errors of immunity with rheumatic features
Source: J Hum Immun. 2026 Aug 10;2(5):e20250218. doi: 10.70962/jhi.20250218 (PMC13455650; doi:10.70962/jhi.20250218)
Supplement: Supplementary file 6 [file jhi_20250218_tables6.docx]

**Table S6. Diagnostic gene panels provided by Kazusa DNA Research Institute**

| **Gene panel category** | **Disease category** | **Target gene** |
| --- | --- | --- |
| **Primary immunodeficiency disorders** | Ectodermal dysplasia | *IKBKG, NFKBIA (IKBA), IKBKB, ORAI1* |
|  | Familial hemophagocytic lymphohistiocytosis | *PRF1, UNC13D, STX11, STXBP2, FAAP24, SLC7A7, LYST, RAB27A, AP3B1, AP3D1, SH2D1A, XIAP (BIRC4)* |
|  | Autoimmune lymphoproliferative syndrome | *FAS, FASLG, CASP8, CASP10, NRAS, KRAS, AIRE, FOXP3, IL2RA, CTLA4, LRBA, STAT3, SH2D1A, IKZF1, PIK3CD, PIK3R1, PRKCD, TNFAIP3* |
|  | Inflammatory bowel disease | *IL10, IL10RA, IL10RB, NFAT5, TGFB1, RIPK1, FOXP3, IL2RA, CTLA4, LRBA, WAS, XIAP (BIRC4), CYBA, CYBB, NCF2, NCF4, TNFAIP3* |
|  | Chronic granulomatous disease | *CYBB, CYBA, NCF2, NCF4, G6PD* |
|  | TLR signaling disorders | *IRAK4, MYD88, TIRAP, IKBKG, NFKBIA, IKBKB, RPSA, NKX2-5, RBCK1* |
|  | Severe combined immunodeficiency (panel 1) | *IL2RG, JAK3, IL7R, RAG1, RAG2, DCLRE1C, ADA, PNP, ZAP70, LIG4, NHEJ1, TBX1* |
|  | Severe combined immunodeficiency (panel 2) | *AK2, CORO1A, FOXN1, PRKDC, PTPRC, STAT5B, ORAI1, STIM1, MAGT1, SEMA3E, POLA1, ATM, CD3D, CD3E, CD247, LAT* |
|  | MHC deficiency | *TAP1, TAP2, B2M, CIITA, RFXANK, RFX5, RFXAP* |
|  | Unclassified immunodeficiency (panel 1) | *TNFSF12, TNFSF13, TNFRSF13B, TNFRSF13C, CD19, CR2, CD81, CD79A, CD79B, PIK3CD, PIK3R1, SH3KBP1, AICDA, PRKCD, ITPKB, NFKB1, IRF2BP2, AP1S3, SH3BP1, REL, EFL1, DNMT3B, ZBTB24, CDCA7, HELLS* |
|  | Unclassified immunodeficiency (panel 2) | *CARD11, BCL10, MALT1, ICOS, IL21R, CD27, CD70, PLCG2, NFKB1, NFKB2, NFKBIA, NFKBIE* |
|  | Neutropenia (panel 1) | *ELANE, HAX1, WAS, CSF3R, SP6A4, CXCR4* |
|  | Neutropenia (panel 2) | *GFI1, G6PC3, SLC37A4, TAZFAZZIN, VPS13B, USB1, JAGN1, CLPB* |
|  | Hyper-IgE syndrome | *STAT3, TYK2, IL6R, ZNF341, ERBIN, TGFRB1, TGFRB2, SPINK5, PGM3, CARD14, DOCK8* |
|  | Chronic mucocutaneous candidiasis | *IL17RA, IL17RC, IL17F, ACT1, TRAF3IP2, AIRE, STAT1, IL12RB1, IL12B, CARD9* |
|  | B-cell deficiency | *BTK, IGHM, IGLL1, CD79A, BLNK, PIK3CD, PIK3R1* |
|  | Complement deficiency (panel 1) | *C3, C1QC, C1QB, C1QA, C1S, C2, C5, C6, C7, C8A, C8B, C8G, C9, MASP2, MBL2* |
|  | Complement deficiency (panel 2) | *SERPING1, F12, PLG, KLKB1, FGA, FGB, FGG* |
|  | Congenital defects in innate immunity (viral susceptibility) | *STAT1, STAT2, IRF7, IFNAR1, IFNAR2, IFNGR1, IFNGR2, TLR3, TBK1, DBR1, IRF8, MAVS, TICAM1, TMEM173* |
|  | Mendelian susceptibility to mycobacterial disease | *IL12RB1, IL12B, IFNGR1, IFNGR2, ISG15, STAT1, CYBB, IRF8, IRF1, TYK2, JAK1* |
|  | Common variable immunodeficiency | *CD40LG, AICDA, CD19, UNG, INO80, PIK3CD, PIK3R1, PTEN, IKBKG* |
|  | IPEX syndrome | *FOXP3, IL2RA, IL2RB, CTLA4, LRBA, STAT5B, FEM1, STAT1, STAT5B* |
|  | Wiskott-Aldrich syndrome | *WAS, ARPC1B, CDC42, WIPF1* |
|  | Congenital poikiloderma | *DKC1, TERC, TERT, TINF2, RTEL1, ACD, WRAP53, PARN, CTCL1, DCLRE1C* |
|  | EBV-associated lymphoproliferative disease | *SH2D1A, XIAP, CD27, RASGRP1, CARMIL2, MAGT1, PRKCD, STK4, ITK, ZAP70, MAGI3, PIK3CD, PI3K, NFKB1, DCL1, CTLA4, PF0, STXBP2, FAS* |
|  | Congenital neutrophil dysfunction | *GATA2, CSF2RA, CSF2RB, IERF1, IRF8* |
|  | Syndromes with combined immunodeficiency | *SAMHD1, RNASEH2A, ZTL3* |
|  | DNA repair disorders | *ATM, MRE11, NBN, RAD50, LIG4, NHEJ1, DCLRE1, PRKDC, DNMT3B, ZBTB24, CDCA7, HELLS, RNF168, MCM4, BLM* |
|  | Autoinflammatory bone disease | *LTGB2, SLC35C1, FEMR3, RASGRP2* |
|  | Congenital neutrophil dysfunction syndrome | *AP3B1, ATP6V1, CTSC, WRD1, MKL1, SLC11A1, CEBPE, G6PD, MPO* |
|  | Shwachman-Diamond syndrome | *SBDS* |
| **Hyper-IgD syndrome** | Hyper-IgD syndrome | *MVK* |
|  | Differential diagnosis for Hyper-IgD syndrome | *MEFV, TNFRSF1A, TNFAIP3, NOD2* |
| **PAPA syndrome (Pyogenic Arthritis, Pyoderma gangrenosum, and Acne)** | PAPA syndrome | *PSTPIP1（CD2BP1）* |
| **Cryopyrin-Associated Periodic Syndrome (CAPS)** | CAPS | *NLRP3* |
|  | Differential diagnosis for CAPS | *NLRC4, PLCG2, NLRP12* |
| **Hereditary Autoinflammatory Syndromes** | NLRC4-associated autoinflammatory disease | *NLRC4* |
|  | Deficiency of Adenosine Deaminase 2 (DADA2) | *ADA2* |
|  | A20 haploinsufficiency | *TNFAIP3* |
|  | Aicardi-Goutières Syndrome | *TREX1, RNASEH2A, RNASEH2B, RNASEH2C, SAMHD1, ADAR, IFIH1* |
|  | Differential diagnosis panel 1 | *MEFV, TNFRSF1A, NLRP3, NLRP12, MVK, PLCG2, NOD2* |
|  | Differential diagnosis panel 2 | *STING1, PSMB8, PSMA3, PSMB4, PSMB9, POMP* |
| **Tumor Necrosis Factor Receptor-Associated Periodic Syndrome (TRAPS)** | TRAPS | *TNFRSF1A* |
| **Nakajo-Nishimura Syndrome (NNS)** | NNS | *PSMB8* |
| **Familial Mediterranean Fever (FMF)** | FMF | *MEFV* |
| **Blau Syndrome** | Blau Syndrome | *NOD2* |

All target gene panels were developed and implemented by the Kazusa DNA Research Institute (Chiba, Japan), using next-generation sequencing (NGS) platforms. Each panel includes the coding exons and flanking splice sites of selected genes. Rare variants (typically defined as allele frequency <1%) were reported. Large structural variants and copy number variants (CNVs) are not assessed in this platform. Somatic mosaicism was reported only if detectable through standard variant calling algorithms.

Abbreviations:

PAPA: Pyogenic Arthritis, Pyoderma gangrenosum, and Acne

CAPS: Cryopyrin-Associated Periodic Syndrome

TRAPS: Tumor Necrosis Factor Receptor-Associated Periodic Syndrome

FMF: Familial Mediterranean Fever

NNS: Nakajo-Nishimura Syndrome
