# Supplementary material for: Multidisciplinary genomic evaluation reveals adult inborn errors of immunity with rheumatic features
Source: J Hum Immun. 2026 Aug 10;2(5):e20250218. doi: 10.70962/jhi.20250218 (PMC13455650; doi:10.70962/jhi.20250218)
Supplement: Supplementary file 7 [file jhi_20250218_tables7.docx]

**Table S7. Variable preprocessing and encoding strategy for MCA and machine learning analyses**

| **Variable** | **Preprocessing / Encoding strategy** |
| --- | --- |
| Family history (FH) | Categorized into 3 = same & similar, 2 = same only, 1 = similar only, 0 = none |
| Infection course | Categorized into 3 = frequent & repeated, 2 = frequent only, 1 = repeated only, 0 = none |
| Serum IgG | Categorized into two groups: Low = < 1350 mg/dL, High = ≥ 1350 mg/dL.  If IgG replacement therapy was present or data were missing, coerced to 300 mg/dL; otherwise, measured value used |
| Lymphocyte count | Categorized into two groups: Low = < 900 /µL, High = ≥ 900 /µL.  Missing values imputed with the cohort median prior to categorization. |
| Age of onset | Categorized into two groups:<40 years (Young), ≥ 40 years (Elder) |
| Autoantibody | Positive = 1, Negative = 0 |
| Other categorical symptoms (rash, arthralgia, chest/abdominal pain, enterocolitis, headache, hearing loss) | Encoded as 1 = presence, 0 = absence |
| Sex | Female = 1, Male = 0 |
| Pre-diagnosis category | ID, AID, or non-ID/AID encoded as categorical variables |
| Course of autoinflammation | Categorized into episodic, periodic, or non-specified (NS) |

Summary table of variable preprocessing and encoding strategy used for MCA and machine learning analyses. For example, family history (FH) was categorized as same & similar, same only, similar only, or none; infection course into frequent & repeated, frequent only, repeated only, or none; serum IgG values during IgG replacement therapy or missing values were coerced to 300 mg/dL.
